# An unusual case of oral mucosal melanoma successfully treated by carbon-ion radiotherapy

**DOI:** 10.1093/omcr/omae108

**Published:** 2024-09-12

**Authors:** Hiroaki Ikawa, Masashi Koto

**Affiliations:** QST Hospital, National Institutes for Quantum Science and Technology (QST), 4-9-1 Anagawa, Inage-ku, Chiba-shi, Chiba 263-8555, Japan; QST Hospital, National Institutes for Quantum Science and Technology (QST), 4-9-1 Anagawa, Inage-ku, Chiba-shi, Chiba 263-8555, Japan

An 81-year-old woman noticed slight discomfort in her oral cavity. She visited her family dentist, and although no obvious abnormality was observed on the dorsal mucosa of the tongue, pigmentation was noted on the sublingual surface and floor of the mouth (Fig. 1a). Therefore, the patient was referred to the oral cancer center. Pathological examination of the biopsy specimen revealed mucosal melanoma (Fig. 2). Relevant medical history included endoscopic submucosal dissection for early gastric cancer (histological type unknown) two years prior, with no evidence of recurrence. Regarding family history, the patient’s grandfather, father, and mother had a history of colorectal cancer. Contrast-enhanced computed and fluorodeoxyglucose-positron emission tomography revealed no distant metastasis of mucosal melanoma. Finally, the initial overall staging of oral mucosal melanoma was T3N0M0. Carbon-ion radiotherapy (CIRT), a particle therapy using carbon ions that was covered by insurance in Japan in 2018, was recommended due to the significant reduction in quality of life expected with surgery. CIRT was administered at its relative biological effectiveness-weighted dose of 57.6 Gy in 16 fractions for 4 weeks. The tumor completely disappeared (Fig. 1b), and follow-up two years after CIRT revealed that the patient’s speaking and swallowing functions were preserved.

**Figure 1:**
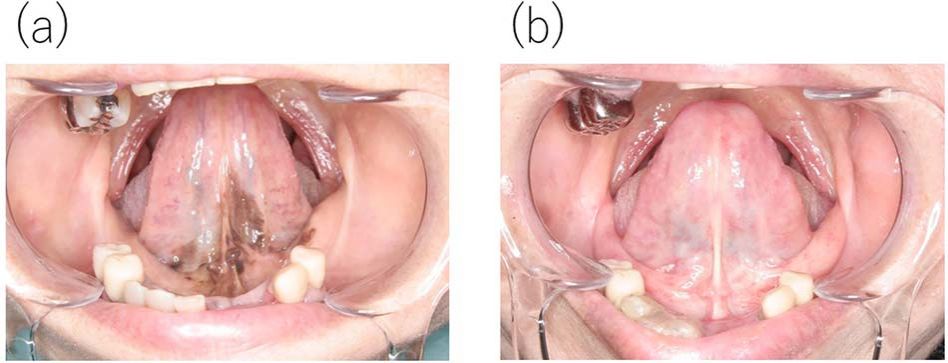
Clinical findings. (**a**) Intra-oral photograph showing the pigmented sublingual mucosa before carbon-ion radiotherapy. (**b**) Intra-oral photograph after carbon ion radiotherapy shows completely disappearance of the mucosal melanoma.

**Figure 2:**
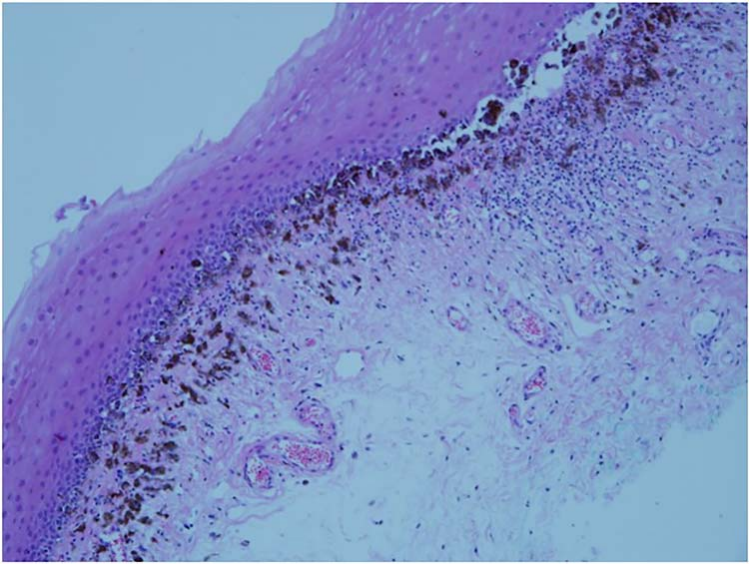
Histopathological findings. Atypical pigmented melanocytes are observed at the junction and invasive nests infiltrate the submucosa.

Most oral mucosal melanomas have few symptoms, resulting in progression and metastasis without recognition, and the prognosis is poor [1]. The standard treatment for resectable oral mucosal melanoma is surgery, and definitive radiotherapy is the treatment option for unresectable cases [2]. To date, outcomes of photon radiotherapy for oral mucosal melanoma have been poor, with a 3-year overall survival rate of 0% [3], and reports on proton-beam therapy mainly for oral mucosal melanoma are lacking. CIRT has a higher linear energy transfer and a greater relative biological effectiveness than photon and proton beam radiotherapies [4]. Therefore, CIRT is a promising treatment option for oral mucosal melanoma, which is relatively radioresistant, with a 5-year local control rate of 93.3% [5].

This case highlights important findings in the diagnosis and treatment of oral mucosal melanoma. First, oral mucosal melanoma can be successfully treated with CIRT. Second, oral physicians must regularly examine not only the dorsal mucosa of the tongue but also the sublingual surface and floor of the mouth for early detection even if there are no symptoms.

## Consent

Informed written consent was obtained from the patient for use of the pictures and publish her case report.

## Guarantor

Hiroaki Ikawa.
